# A small area analysis of acute exposure to temperatures and mental health in North Carolina

**DOI:** 10.1007/s00484-025-02858-y

**Published:** 2025-02-04

**Authors:** Sophia C Ryan, Luke Wertis, Margaret M. Sugg, Jennifer D. Runkle

**Affiliations:** 1 Department of Geography & Planning, Appalachian State University, Boone, NC USA; 2 North Carolina Institute for Climate Studies, North Carolina State University, Raleigh, NC USA

**Keywords:** Temperature, Mental health, Behavioral disorders, Small-area analysis, DLNM

## Abstract

Increasing evidence suggests that temperatures adversely impact mental and behavioral disorders (MBD). This study explores the effects of temperatures on mental health outcomes using over 5.9 million MBD-related emergency department (ED) visits across three geographical regions of North Carolina (i.e., Mountains, Piedmont, and Coast) from 2016 to 2019. A distributed lag non-linear model (DLNM) with a generalized linear model and quasi-Poisson distribution adjusted for humidity, long-term seasonal time trends, and day of the week examined the acute impact (i.e., 7-day) of temperature on daily MBD-related ED visits at zip code tabulation area (ZCTA) locations. Results were pooled at the region and state levels and reported in reference to the median temperature using a case-time series design for the analysis of small-area data. Stratified analyses were conducted for age, sex, and specific mental-health related ED visits (substance use, mood disorders, anxiety disorders). At the state level, we found significant positive associations between high temperatures (97.5th percentile) and an increase in relative risk (RR) for total MBDs (RR:1.04, 95% CI,1.03–1.05) and psychoactive substance use (RR:1.04, 95% CI, 1.02–1.06). Low air temperatures (2.5th percentile) only increased risk for the elderly (i.e., 65 and older) and predominantly white communities (RR: 1.03, CI: 1.03–1.05). During high temperatures (97.5th percentile), majority-white communities (RR:1.06, CI: 1.01–1.10) and low-income communities had the highest risk for MBDs (RR: 1.05, CI: 1.03–1.07). Our findings suggest there is a positive association between exposure to high temperatures and increased MBD-related ED visits, modified by patient age and place-based sociodemographic (ie., race and income) context.

**Supplementary Information:**

The online version contains supplementary material available at 10.1007/s00484-025-02858-y.

## Significance statement

This research investigates the impact of air temperatures on mental and behavioral disorders, focusing on residents across North Carolina. By analyzing over 5.9 million emergency room visits, we identify the effects of temperature on different demographic and mental health subgroups for each of the three geographic regions. These results further the understanding of the association between temperatures and mood-behavioral disorders and provide a new understanding of how this association changes across community income and racial segregation measures.

**Supplementary Information:**

The online version contains supplementary material available at 10.1007/s00484-025-02858-y.

## Introduction

Mental and behavioral disorders (MBDs) encompass a wide range of mental health concerns, including mood disorders, anxiety disorders, psychoactive substance use disorders, schizophrenia, and intellectual disabilities. MBDs have increased substantially in the last decade (Duffy et al. 2019; Eisenberg 2019). This increase has been most pronounced among young people (Duffy et al. 2019; Eisenberg 2019) and can be attributed in part to increased social media use (Sadagheyani and Tatari 2021), increasing social stressors (Mossakowski 2014), and the COVID-19 pandemic (Hamza Shuja et al. 2020; Nochaiwong et al. 2021; Olff et al. 2021; Torales et al. 2020). While the pathways in which poor mental health outcomes develop are complex, a growing body of evidence, including reviews (Ebi et al. 2021; Palinkas and Wong 2020; Thompson et al. 2018), time-series analyses (Sugg et al. 2019; Wertis et al. 2023), and distributed lag non-linear model analyses (Wang et al. 2014; Yoo, Eum, Gao, et al., 2021), has linked poor mental health prevalence with environmental exposures, such as extreme heat, tropical cyclones, and wildfires. These exposures are hypothesized to increase stress loads (Bell et al. 2018), cause lasting trauma (Berry et al. 2010), and increase impulsivity and risky behaviors (Tiihonen et al. 2017). Extreme events, like high temperatures, are expected to increase (Kunkel et al. 2020), stressing the importance of research to understand how exposure influences patterns of mental health.

Past research has widely used distributed lag non-linear models (DLNMs) to investigate complex exposure-outcome relationships at different lag periods (e.g., up to seven days following exposure) to see how the risk of an outcome varies with exposure (Gasparrini et al. 2010). This method provides a flexible matrix to assess non-linear exposure-response relationships at different lag periods (e.g., 21 days after exposure). For example, DLNM analyses have examined extreme temperatures and mental health (Minor et al. 2023; Yoo, Eum, Gao, et al., 2021; Yoo et al. 2021), heat and mortality (Dutta et al. 2020; Yi and Chan 2015), temperature and heat-related illness (Bai et al. 2014), and air pollution and health (Luo et al. 2022; Yang et al. 2022).

Recent work employing DLNMs suggests that exposure to extreme temperatures can be harmful to mental health (Wang et al. 2014; Yoo, Eum, Gao, et al., 2021; Yoo et al. 2021). Past research in New York, USA, found exposure to extreme heat (temperatures above the 97.5th percentile) and cold (temperatures below the 2.5th percentile) were associated with a higher risk of poor mental health (Yoo et al. 2021). In addition, extreme heat was associated with a higher prevalence of substance use disorders, dementia, schizophrenia, and mood and anxiety disorders (Yoo et al. 2021), and this association did not vary based on demographic factors (i.e., age, race, sex). In Canada, Lavigne et al. (2023) and Wang et al. (2014) also found extreme heat (temperatures above the 97.5th percentile, 99th percentile, respectively) was associated with a higher prevalence of mental and behavioral disorders, substance use disorders, and dementia, but in contrast, extreme cold (temperatures below the 2.5th percentile) was associated with a lower prevalence of MBD; suggesting the association between extreme temperatures and mental health outcomes may vary with geographic context. Lavigne et al. (2023) further highlight disparities in extreme temperature exposure and mental health risks, indicating that neighborhood deprivation (as measured by the Material and Social Deprivation Index) and pre-existing mental health conditions may increase an individual’s risk of poor mental health following exposure to extreme temperatures. Research in the Southeastern United States using DLNMs in select urban counties suggests that extreme temperature exposure is weakly associated with poor mental health outcomes in Minor et al. (2023); whereby urban counties (e.g., Buncombe, Mecklenburg, Guilford, Wake, Pitt, New Hanover) characterized by greater temperature variability had a stronger association with temperature and mental health prevalence. This work, like previous DLNM studies (e.g., Bai et al. 2014; Dutta et al. 2020), is limited to populated locations with high sample sizes (i.e., urban areas), however, a new DLNM extension allows for analysis across small geographic regions, with smaller population sizes, as a small-area approach (Gasparrini 2022). This methodological advancement allows control for both time-invariant and time-varying confounding as well as the assessment of complex temperature-health associations across finely disaggregated geographic contexts where underlying contextual factors may modify the temperature-mental health association (Gasparrini 2022).

A large body of evidence suggests both individual and contextual factors contribute to health disparities, with community economic context recognized as one of the biggest predictors of poor health outcomes at the community-level (Pickett and Pearl 2001; Drukker et al. 2003). In North Carolina, persistent mental health disparities exist at the community-level (Ryan et al. 2022; Sugg et al. 2021, 2022; Ulrich et al. 2023), driven in part by place-based factors, including rurality and neighborhood environments with lower socio-economic status (Ryan et al. 2022; Sugg et al. 2021). As a key social determinant of health (*CDC*,* 2024*), economic instability is associated with increased incidence of poor mental health at the individual and community-level (Drukker et al. 2003; Ryan et al. 2022; Sugg et al. 2021). Evidence further suggests low income communities are less likely to have mental health care facilities, partially contributing to disproportionate mental health burdens (Cummings et al. 2017). Mental health disparities are also co-occurring with higher temperatures, and climate change is projected to change temperature patterns with an additional two to three weeks per year above 95oF expected in North Carolina by 2040 (Kunkel et al. 2020).

This retrospective analysis aimed to investigate the association between temperature exposure and mental health-related emergency department visits in North Carolina, USA, at the ZCTA (ZIP Code Tabulation Areas) level from 2016 to 2019. Using the small-area DLNM, we extend previous work to analyze the temperature-mental health association across an entire state using a fine geographic scale (e.g., ZCTA). We further stratified the analysis to consider physiographic regional differences (e.g., mountains, piedmont, coastal plain) and geographic and place-based contexts (e.g., predominantly low income vs. predominately high income neighborhoods). We performed a subgroup analysis to determine how the association between mental health and temperature changes across demographic variables (i.e., age and sex) and MBD subgroups (i.e., psychoactive substance use, mood, and anxiety). Unlike previous work examining temperature-mental health (e.g., Basu et al. 2018; Yoo et al. 2021), we extend our analysis to include community measures of racial and income segregation using well-established public health metrics (Krieger et al. 2016), which have been shown to have a strong association with mental health (Ryan et al. 2023; Sugg et al. 2022). Our results will further the understanding of how exposure to temperature can impact community mental health burden across different subgroups and geographic locations.

## Materials and methods

### Study area

**Fig. 1:**
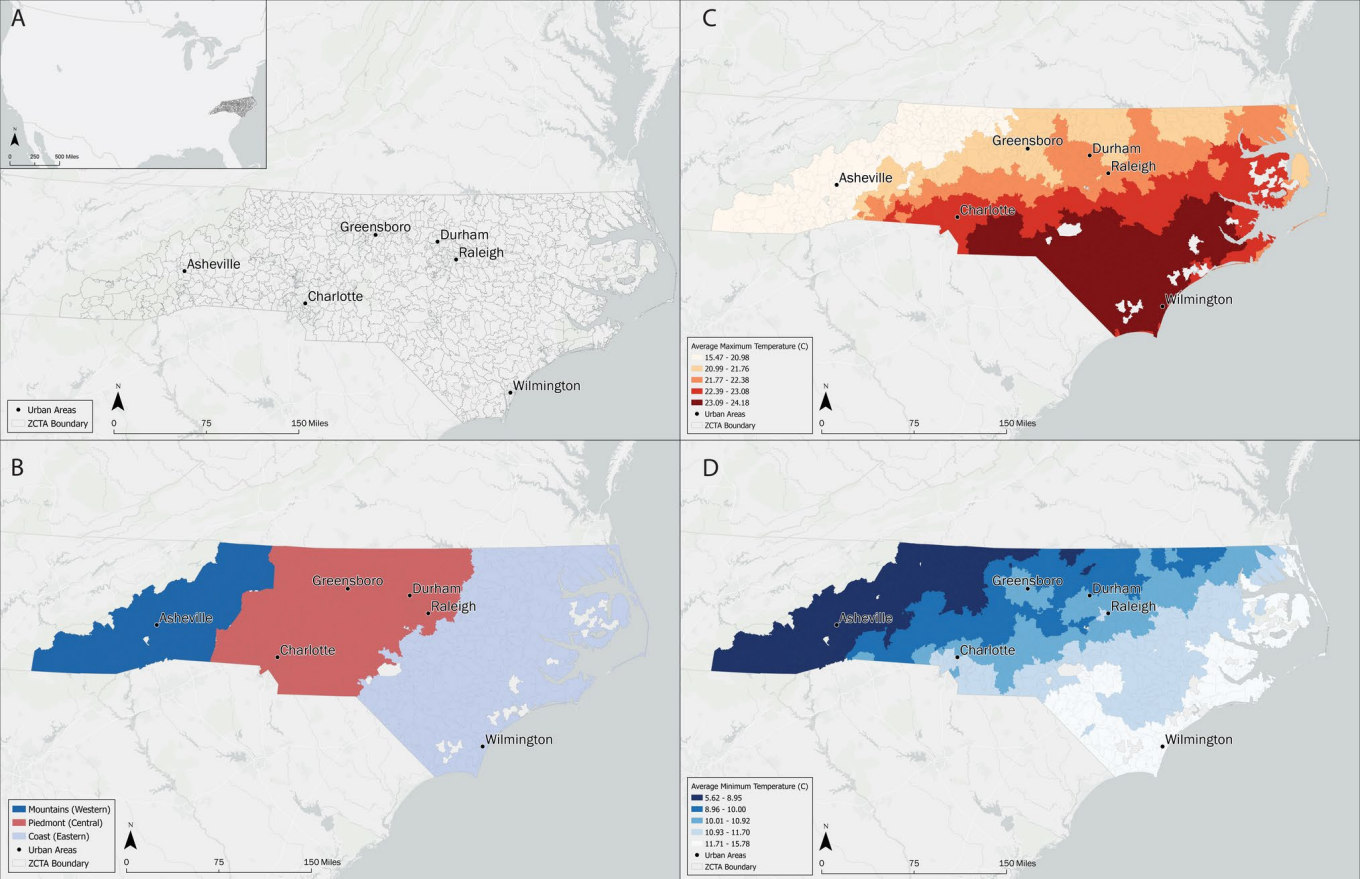
Map panel depicting (**A**) North Carolina’s relative location within the United States, (**B**) the three geographic boundaries of North Carolina including the western Mountains, central Piedmont and the eastern Coastal Plain, (**C**) average maximum temperature (C^o^) and (**D**) average minimum temperature (C^o^)

North Carolina (NC) is a state in the southeastern United States (Fig. 1). As of the 2020 US Census, NC has a population of 10.8 million and is characterized as 60.7% non-Hispanic white, 22.1% non-hispanic Black or African American, 1.6% American Indian and Alaska Native, 3.7% non-Hispanic Asian, 2.9% Other Race and Multirace, and 11.4% Hispanic or Latino (US Census 2022). Characterized by a humid subtropical climate, NC has three physiographic regions defined by the North Carolina Department of Environmental Quality: the mountains (west) the piedmont (central) and the coast (east), which represent eight separate climate zones (Fig. 1) (NC DEQ 2018). These regions have been considered in previous studies examining climate and health associations in North Carolina, revealing varied climate-health patterns (Choi et al. 2022; Elcik et al. 2017; Sugg et al. 2016).

### Data

#### Study population

Daily emergency department (ED) admission data for North Carolina (NC) were obtained from the Shep’s Center for Health and Human Services Research (January 1, 2016- December 31, 2019); this time period was determined based on the change from ICD-9 to ICD-10 codes in October 2015 (US Department of Labor 2015), leading to a classification change in several mental health-related codes (UNC Sheps Center 2022). We elected not to include data during the COVID-19 pandemic due to administrative and behavioral changes in ED visits, limiting our time period to 2016 to 2019. In this study, we identified daily visits for mental disorders based on the patient’s diagnosis codes using the International Classification of Disease Tenth Revision (ICD-10: F00-F99). Our grouping and selection of records are largely based on published studies (Trang et al. 2016; Wang et al. 2014; Yoo et al. 2021b). We classified and grouped mental disorders into three specific diseases: psychoactive substance use (ICD10: F10-F19), mood disorders (ICD10: F30-39), and anxiety disorders (ICD10: F40-49). A time series of daily case counts for each MBD-related visit across NC at the ZCTA level was constructed for January 1, 2016, to December 31, 2019. The ED data contains line-level information on admission date, discharge date, 5-digit zip code of residence, and demographic information (age in years (0–25, 26–49, 50–64, 65+) and sex (male, female) of individuals). The ED is a major entryway to the mental health system for US populations (Gill et al. 2017) and has been used to analyze previous temperature-mental health associations (Basu et al. 2018; Yoo et al. 2021b).

For this study, the unit of analysis was the Zip-Code Tabulation Area (ZCTA) level, with individual ED data converted from zip code to ZCTA when applicable (AAFP 2022). In NC, there are 802 ZCTAs, which are US Census Bureau spatial geographies corresponding to US Postal Service zip codes. ZCTA is the finest spatial resolution available for this health dataset (Fig. 1). ZCTAs were categorized into the three geographical regions of North Carolina: Mountains, Piedmont, and Coastal Plains. The month of the year, day of year and day of the week were notated in the data set and incorporated into the final models.

#### Weather data

Daily gridded raster temperature data at 4 km resolution was obtained from the PRISM Climate Group from January 1, 2016, to December 31, 2019 (PRISM 2023) (Fig. 1); the raster was aggregated to the ZCTA level by taking a weighted mean of daily average temperature (TAVG) (°C) and dewpoint temperature (°C) for all grid points within a ZCTA. Weighted means were spatially aggregated using an area-weighted approach and linked to individual-level ED data using the zip code of patient residence. In addition to the metrics obtained by PRISM, relative humidity (RH) (%) was calculated using the weathermetrics package (Anderson et al. 2016). The inclusion of RH, as a confounder, is similar to other temperature-health analyses that use the DLNM with temperature as the main focus and RH as a potential confounder in the association (Almendra et al. 2019; Lee et al. 2018; Peng et al. 2017). Ideally, for studies focused on humidity measures, other metrics like absolute humidity or dewpoint temperature would be integrated. R 4.2.0 was utilized to perform this raster analysis using terra (Hijmans et al. 2024) at the ZCTA level (R Core Team 2022).

#### Socio-demographic data

Community-level socio-demographic information was operationalized using the Index of Concentration at the Extremes (ICE) metrics (Krieger et al. 2016). Metrics were calculated using race and income data from the 5-year estimates of American Community Survey (2016) for each ZCTA in North Carolina (US Census 2018). ICE metrics are widely used in public health monitoring to capture extremes of economic and residential segregation at the neighborhood level (Conner et al. 2010; Krieger et al. 2016) and are well-established as a spatial metric of measures like structural racism (Yearby et al. 2022) and income inequality that can be computed at different geographic scales and reduces issues of multicollinearity. The ICE income metric captures the number of individuals in the 80th percentile of income subtracted from the 20th percentile, divided by the total population with a known income. The ICE race metric is calculated from the ratio of white individuals to Black individuals and represents a comparison between majority Black compared to majority white communities at each extreme (Krieger et al. 2016). The ICE metrics range from − 1 (i.e., majority low income, majority Black) to + 1 (i.e.,. majority high income, majority white) (Krieger et al. 2016) and were operationalized as quantiles (Krieger et al. 2016; Wallace et al. 2019). A map of ICE values is shown in Supplemental Fig. 1.

#### Statistical analysis

We used a small-area-analysis approach of the DLNM to examine the association between temperature and MBD ED visits at the community level (i.e., ZCTA) (Gasparrini 2022). The small-area-analysis approach allows for the inclusion of multiple spatial locations (e.g., over 800 ZCTAs) rather than a single city; this method is an extension of the original DLNM approach, initially proposed as a case time series design on short-term associations with time-varying exposures (Gasparrini 2022). The small-area analysis allows for the investigation of large health and exposure (i.e., temperature) datasets, where outcomes were obtained at a small geographic scale (Gasparrini 2022). Furthermore, aggregating health and temperature data at finer spatial scales reduces the likelihood of ecological fallacies, which large scale aggregation studies are often prone to (Gasparrini 2022). In addition, unlike two-stage designs developed for multi-city investigations (Zafeiratou et al. 2019), this extended case time series approach does not have the same limitation of estimation issues in the presence of small sample sizes due to small-scale geographic units or rural locations (Gasparrini 2022).

The small-area DLNM combined with a generalized nonlinear model (gnm) was performed due to prior literature demonstrating a non-linear and delayed (e.g., typically 3 to 7-day lag) relationship between temperature and MBD-related ED visits (Crank et al. 2023; Gasparrini et al. 2010, 2022; Peng et al. 2017; Yoo et al. 2021b). Unlike other DLNM analyses, the case time-series design for small areas created by Gasparrini (2022) allows for the flexibility to include small population sizes and geographic units and therefore builds upon prior temperature-mental health association that has been limited to high population locations. In this analysis, each of the three regions of North Carolina were stratified by the ZCTA that comprises that region and were analyzed individually (Fig. 1). This regional approach has been applied in other health-temperature studies (Elcik et al. 2017; Kovach et al. 2015; Sugg et al. 2016), which demonstrate significant variability in the temperature-health association across each geographic region. In each region, a DLNM fitted with a gnm was applied as a quasi-Poisson distribution with a lag period of seven days in order to establish the associations between temperature and the relative risk (RR) of increased ED visits over an acute time period. DLNM is able to characterize the non-linear exposure-response relationship at varying delayed exposure times (Gasparrini 2011). For this analysis, the region-specific and state-specific temperature-ER visit association for MBDs were calculated. The model was: 

1 $$logE(Yijk)=\alpha+cb(Temp_{ik})\:+\:ns(RH_{ik},df1)\:+\:ns(doy_i,knots).factor(year_k)+\sum\nolimits^7l=_1\beta l.I(DOWik=l)+\gamma_{ZCTAj,yeark,monthm}$$

Where *E(Yijk)* was the expected ED visits related to MBDs for observation *i* at location (i.e., ZCTA) *j* at time *k* as a logarithmic function of the intercept (*α*); *cb()* denoted the cross basis function for temperature (daily temperature), three-knot values in the lag space of the cross basis-function defining the exposure-response were set equally spaced in the log scale of lags for more flexible lag effects at shorter delays and a lag-response (one knot at lag 1 over the lag period 0–7) relationship (Yoo et al. 2021a, b; Gasparrini 2011). *ns()* denoted the natural cubic spline, which was applied to relative humidity and the time trend. Longer term time trends were inlcuded and consist of the natural cubic splines of the day of the year with degrees of freedom of 3 and year indicators to model differential seasonal effects. The day of the week (*DOW*_*t*_ ) was included to adjust for short-term (daily and weekly) human behavior patterns. Both DOW and  longer term time trends were used as covariates for the temperature and relative humidity variables (Dominici 2004). The degrees of freedom (*df*) for the predictors were set; *df*_*1*_ = 2 for relative humidity to adjust for humidity in the temperature-mental health association and *df*_*2*_ = 3 for the time trend to model for the season and long-term time trends. These degrees of freedom across model parameters were identified based on previous studies (Yoo et al. 2021a, b; Gasparrini 2011; Crank et al., 2023; Peng et al. 2017) and then tested for the best-fitting model based on quasi-AIC (Guo et al. 2011).

We calculated the pooled cumulative effect of temperature for the relative risks (RR) and 95% confidence intervals (CIs) of the MBD hospital admission visits at the 2.5th and 97.5th percentile of average temperature relative to the median average temperature for the cumulative lag effect of 0–7 days. Temperature percentiles are calculated based on the time series for each ZCTA from 2016 to 2019. Analysis was conducted using the dlnm (Gasparrini 2011) and mixmeta (Gasparrini and Sera 2021) packages for distributed lag models and meta-analyses, respectively, in R version 4.2.0.

#### Subgroup analysis

We also conducted a stratification analysis using the same methodology for temperature effects on multiple subgroups, stratifying by individual-level factors obtained from the ED data and community-level (i.e. ZCTA) race and socioeconomic status. Based on individual-level variables provided in the ED data (UNC Sheps Center 2022), data was stratified by (1) mental disorder subgroups: psychoactive substance use, mood, and anxiety, (2) by sex (female and male), and (3) by age groups (below 25 years, 26–49 years, 50–64 years, and above 65 years). At the community level, data was stratified at the ZCTA-level to compare temperature-mental health risk based on community-level income: ICE: Income (Q1: High Income, Q4: Low Income) and community-level race: ICE: Race (Q1: Majority White, Q4: Majority Black). The stratification of models by individual race metrics from the ED data was not conducted due to low sample sizes. All models except those focused on specific mental health outcomes were analyzed using all mental health ED visits (ICD-10: F00-F99).

## Results

### Summary of environmental variables and ED visits

Between January 1, 2016, and December 31, 2019, there were 5,949,100 MBD-related ED visits in North Carolina. The characteristics of ED visits and physical environmental conditions are summarized in Table 1. The most common diagnosis for MBD-related ED visits was attributed to psychoactive substance use (4,111,061), followed by anxiety disorders (1,451,168) and mood disorders (1,421,839). The age group of 26 to 49 years old (639,997, 30.7%) represented the highest percent, followed by 50 to 64 years old (521,077, 25.0%), age 65 or older (497,877, 23.9%), and 25 or younger (423,724, 20.3%), respectively. Females represented the majority of visits (3,045,395, 51.2%). These outcome distributions are similar to population estimates, with 51% of the NC population reporting Female, 24% of the NC population 65 and older and 37% of the NC population under age 20 (US Census 2022).

During the study period, the daily average temperature in the study region ranged from − 15.37 °C to 31.79 °C with a mean of 16.34 °C and a standard deviation of 8.89 °C. North Carolina has four distinct seasons, with both summer and winter temperatures varying considerably between regions. For the Mountain (western) region of North Carolina, the annual mean temperature was above 13.5 °C, with a cold winter and cooler summer temperatures compared to the other two regions. The Piedmont (central) region of North Carolina was much warmer than the Mountains and cooler than the Coastal region of North Carolina in the summer, with an annual daily temperature of 16.31 °C. Lastly, the Coastal (eastern) region of North Carolina had a milder winter temperature than the other two regions, partially attributed to the warm waters of the Gulf Stream and low elevation, with an annual mean temperature of 17.61 °C. The high and low temperatures in this region were calculated based on time-series of each ZCTA using the 97.5th and 2.5th percentiles, a common approach for DLNM-based analysis (Yoo et al. 2021a, b; Lavigne et al. 2023).

**Table 1:** Sociodemographic information for ED visits across the three geographical regions of North Carolina and the entire state from January 2016 to December 2019

	Mountains	Piedmont	Coast	North Carolina
**Total Population**	1,099,538	5,942,142	2,803,653	9,845,333
**Total Mental and Behavioral** **Disorder ED**^**1**^**Visits**	*n* = 760,*040*	*n* = 3,*391*,*613*	*n* = 1,*797*,*447*	*n* = 5,*949*,*100*
**Specific mental disorders (n=)**				
*Psychoactive Substance Use*	533,235	2,335,924	1,241,902	4,111,061
*Mood*	208,913	803,011	409,915	1,421,839
*Anxiety*	226,600	806,742	417,826	1,451,168
**Age group (%)**				
*0–25*	18.9	21.2	19.6	20.3
*26–49*	31.6	29.9	31.6	30.7
*50–64*	24.9	24.8	25.4	25
*65+*	24.6	24	23.4	23.9
** Sex (%) **				
*Male*	48.3	48.9	49	48.8
*Female*	51.7	51.1	51	51.2
**ICE**^**2**^**Race (%)**				
*Q1: Majority White*	96.2	53.7	32.3	53.9
*Q2: Mixed Race Majority White *	3.7	24.3	43.7	27.5
*Q3: Mixed Race Majority Black *	0	14.6	17.3	13.5
*Q4: Majority Black *	0	7.5	2.7	5.1
**ICE Income (%)**				
*Q1: Lowest Deprivation (Majority high-income)*	0.2	6.5	1.4	4.1
*Q2: Middle Low Deprivation*	5.3	19.4	9.0	14.4
*Q3: Middle High Deprivation*	44.1	45.0	51.8	46.9
*Q4: Highest deprivation (Majority low-income)*	50.3	29.1	37.3	34.5
**Weather**				
*Average temperature*,* °C*	13.53 (8.52)	16.31 (8.98)	17.61 (8.68)	16.34 (8.89)
*Relative Humidity*,* %*	70.21 (15.23)	67.59 (14.60)	71.70 (12.98)	69.74 (14.21)

^1^Emergency Department ^2 ^Index of Concentration at the Extremes

### Distributed lagged effects of temperature on ED visits

**Fig. 2:**
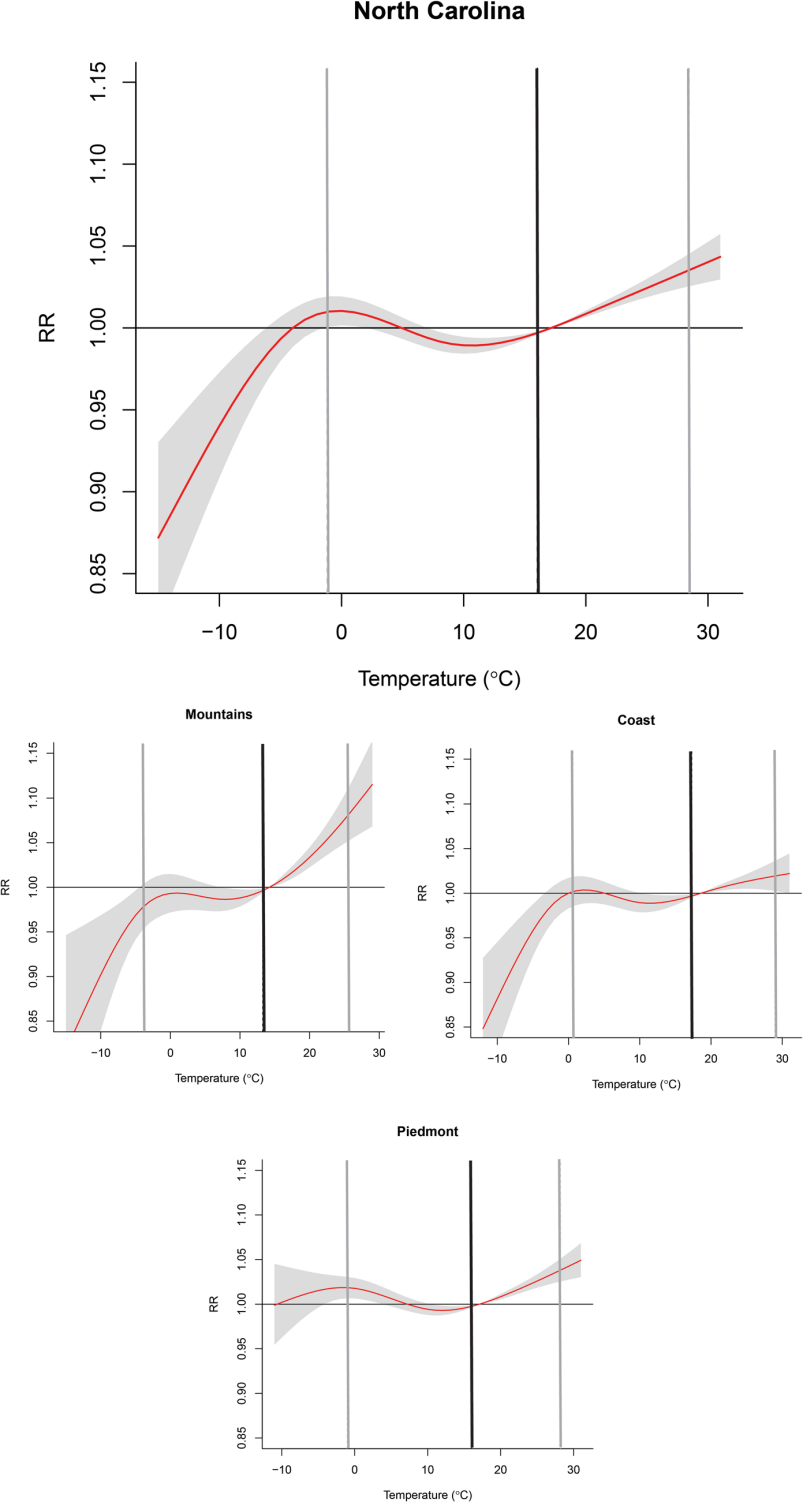
The overall cumulative effect of temperature for all mental and behavioral disorder-related ED visits with an extended lag period (Lag 0–7) across North Carolina (top panel). The pooled effect of the three different geographic regions in North Carolina of temperature for all mental and behavioral disorder-related ED visits (bottom panels). Risks were derived relative to the median temperature. The red line indicates the relative risk, with the shaded area representing the 95% confidence intervals (CI), grey lines representing the 2.5th and 97.5th temperature percentile, and the black line representing the optimal emergency room visit temperature. The optimal emergency room visit temperature was defined as the temperature corresponding to the minimum risk of emergency department visits

Results indicated that there was a significant positive association between MBD-related ED visits and the pooled cumulative effect of daily average temperature across North Carolina (Table 2) for high temperatures (97.5th percentile, RR = 1.04; 95% CI: 1.03–1.05) and no effect for low temperatures (2.5th percentile: 1.01; [1.00–1.02]) (Fig. 2). We observed a significant increase in MBD-related ED visits at the 97.5th percentile of temperature for the Mountains (1.09, [1.06–1.12]), Piedmont (1.04, [1.03–1.05]), and Coast region (1.02, [1.01–1.04]). An increased risk for exacerbated MBDs at the 2.5th percentile temperature was only observed for the Piedmont region (1.02, [1.01–1.03]) (see Supplemental Table 1 for region-specific RR and 95% CI).

### Subgroups

**Fig. 3:**
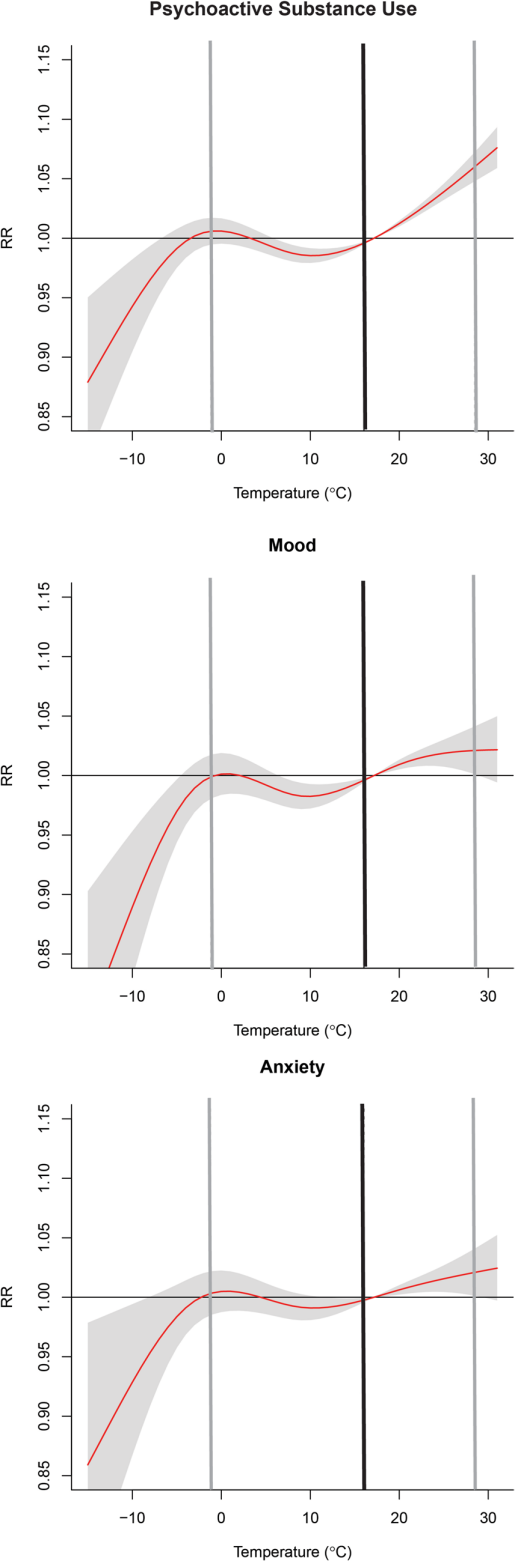
The overall cumulative effect of average temperature for (1) psychoactive substance use, (2) mood, and (3) anxiety-related ED visits with an extended lag period (Lag 0–7) across the state of North Carolina. The optimal emergency room visit temperature was defined as the temperature that corresponded with the minimum risk of emergency department visits. The red line indicates the relative risk, with the shaded area representing the 95% confidence intervals (CI), grey lines representing the 2.5th and 97.5th temperature percentile, and the black line representing the optimal emergency room visit temperature

We examined the pooled cumulative effect of temperature on MBD-related ED visits across North Carolina for multiple subgroups of mental and behavioral disorders (Fig. 3). Only psychoactive substance use exhibited a significant positive RR at the 97.5th percentile of temperature (a null effect was observed for low temperatures (i.e., 2.5th percentile) across subgroups of mental health conditions. Similarly, a significant increase in risk for psychoactive substance use was observed for all three regions, and only a positive association between extreme heat and elevated ED visits for mood and anxiety disorder for the Mountain (western) region (Supplemental Table 1). For low temperature, we observed heterogeneity in the exposure-response relationship across regions. The RR for psychoactive substance use was significantly lower in the Mountains (0.97, [0.94–0.99]) and higher in the Piedmont (1.02, [1.01–1.04]). A lower risk of mood-related ED visits was observed in the Mountains, and no association between cold exposure and mood or anxiety disorders was observed for the Piedmont (central) and Coastal (eastern) regions. The most extreme cold temperatures (i.e., less than 2.5th percentile) had a protective effect in the Mountains and Coastal regions (Fig. 2).

Table 2 demonstrates stratified results by high and low temperature for effect modifiers, including sex, age group, ICE: Race, and ICE: Income. Males were more sensitive to high temperatures (1.05, [1.03–1.06]), but females also experienced an elevated risk of MBD during periods of high temperatures. Young people (< 25 years) had a lower risk of an MBD visit following acute exposure to high temperatures, while older adults experienced a higher risk of MBD-related ED visits during low temperatures (1.03: [1.01–1.05]). We also observed a higher risk of psychiatric ED visits for persons aged 26 to 49 years (1.06, [1.05–1.08]) and 50 to 64 years (1.06, [1.04–1.08]) in response to high temperatures.

Meanwhile, the association between high temperature exposure and MBD-related ED visits differed across quantiles of ICE: Income, the middle-high and highest levels of income deprivation were characterized by higher but generally similar risks. The relative risk was also highest for the extremes of ICE: Race during high temperatures, with high RRs noted for both majority white (e.g., Q1) and majority Black locations (e.g., Q4). For high and low temperatures, predominately white locations (e.g., ICE Race Q1) had the highest relative risk (Low: 1.05, [1.01–1.09], High: 1.06, [1.01–1.10]).

**Table 2:** Relative risk at the low temperatures (2.5th percentile) and high temperatures (97.5th percentile) compared to median temperature (50th percentile) for total MBD-related ED visits in North Carolina (2016–2019) across a seven-day lag period

	North Carolina	
	Low Temperatures (2.5th percentile)	High Temperatures (97.5th percentile)
Mental and Behavioral Disorders		
*Total*	1.01 (1.00–1.02)	1.04 (1.03–1.05)
*Substance*	1.00 (0.98–1.02)	1.04 (1.02–1.06)
*Mood*	1.00 (0.98–1.02)	1.02 (1.00–1.04)
*Anxiety*	1.00 (0.98–1.02)	1.02 (1.00–1.04)
** Sex **		
*Male*	1.01 (1.00–1.02)	1.05 (1.03–1.06)
*Female*	1.00 (0.99–1.01)	1.03 (1.02–1.04)
**Age group**		
*Below 25*	0.97 (0.95–1.00)	0.97 (0.94–0.99)
*26–49*	1.00 (0.99–1.02)	1.06 (1.05–1.08)
*50–64*	1.02(1.00–1.04)	1.06 (1.04–1.08)
*Above 65*	1.03 (1.01–1.05)	1.00 (0.98–1.02)
**ICE**^**1**^ **Race**		
*Q1: Majority White*	1.05 (1.01–1.09)	1.06 (1.01–1.10)
*Q2: Mixed Race Majority White *	1.02 (0.99–1.04)	1.02 (1.00–1.05)
*Q3: Mixed Race Majority Black *	1.00 (0.99–1.02)	1.03 (1.02–1.05)
*Q4: Majority Black*	1.01 (1.00–1.03)	1.04 (1.03–1.06)
**ICE Income**		
*Q1: Lowest Deprivation (Majority high-income)*	0.99 (0.95–1.03)	0.96 (0.92–1.00)
*Q2: Middle Low Deprivation*	1.01 (0.99–1.03)	1.03 (1.00–1.05)
*Q3: Middle High Deprivation*	1.02 (1.00–1.03)	1.04 (1.03–1.06)
*Q4: Highest Deprivation (Majority low-income)*	1.01 (0.99–1.03)	1.05 (1.03–1.07)

^1^Index of Concentration at the Extremes

## Discussion

This analysis leveraged DLNMs, a widely used exposure-time-response model, to examine the mental health effects of temperature in North Carolina between January 2016 and December 2019. Unlike previous work with the DLNM (Crank et al. 2023; Minor et al. 2023), we extended the case time series design proposed for individual-level analyses and common to city-level analysis, to data aggregated over small geographic areas (Gasparrini 2022). Our analysis found that at the state level, short-term exposure (i.e., up to seven days) to high temperatures was associated with increased risk for total psychiatric burden and psychoactive substance use. During high temperatures, males, working-age adults (e.g., 26–49, 50–64) and individuals residing in low-income, majority Black or majority white communities were at higher relative risk for an MBD-related ED visit. Older adults (65 years+) and persons who reside in majority white communities are at a higher relative risk for any psychiatric disorder following exposure to acute periods of low temperatures. These findings support the need to consider individual and place-based context, notably systemic racial and economic factors, when investigating complex population-level associations between environmental exposures and mental health conditions.

Our findings suggest there is a positive association between short-term exposure to high temperature (i.e., up to seven days) and increased MBD-related ED visits, which is consistent with earlier studies employing the DLNM showing increased relative risks for MBD-related ED and hospital admissions with high temperature exposure (e.g., Bundo et al. 2021: 4% increase per 10 °C increase in average air temperature; Yoo et al. 2021; increasing risk above 27.07∘C; Crank et al. 2023; increased risk above 30∘C). However, results across studies show significant variation in higher risk at 0-day lag to three or seven days after exposure and vary based on the analysis’s temperature thresholds or geographic scale. Our study is unique in that our analysis was conducted at the ZCTA scale, a smaller unit than the county but available across an entire US state. ZCTA-scale health analyses are more sensitive to community-level health trends and spatial patterns, as compared to county-level analyses (Jones and Kulldorff 2012).

Results from prior studies linking low temperatures with increased risk for MBD-related emergency visits have been mixed; some studies have identified a delayed effect of 14 days (Yoo et al. 2021), and others showed no association between ED visits for mental disorders and population-level exposure to low temperatures (Peng et al. 2017; Wang et al. 2014). Additional studies have suggested that low temperatures (−2 °C,10th percentile) may be protective for MBD in temperate Northern China among the general public (Gao et al. 2023); while others have found low temperatures (< 12.72 °C) increased the risk of depression among middle-aged and older adults in China (Jin et al. 2023). Jin et al. (2023) hypothesize that different populations are vulnerable to high and low temperatures, with older adults and individuals living in the south of the country potentially experiencing worse outcomes following exposure to nighttime low temperatures. Limited US-based studies have identified an elevated risk of MBD-related ED visits among older adults following periods of low temperatures and cautioned that this group might be more sensitive to low variability (Yoo, Eum, Gao, et al., 2021). Our study corroborates this finding, with older adults experiencing the greatest increase in MBD in response to low temperatures, further stressing the need for place-based and population-specific analyses to understand differential vulnerability to temperature.

Our findings revealed a consistent relationship between acute exposure to high temperature and psychoactive substance use at the state and regional levels (i.e., Mountains, Piedmont, Coastal). Individuals with substance misuse may ingest substances that impair their natural thermoregulation response to temperature. Examples include opiates that interfere with skin vascularization or alcohol, which depresses the central nervous system (Wang et al. 2014). In addition, high temperature can influence psychoactive substance use through thermoregulation mechanisms, including dehydration, which affects drug concentration in the bloodstream, and impaired cognitive function, which may promote risky behaviors (Cusack et al. 2011). While a growing literature has linked climate change with excess mental health burden (Charlson et al. 2021), population-level exposure to more frequent or intense climate hazards (e.g., increasing ambient temperatures, more frequent and intense heat waves) may directly or indirectly be associated with new or exacerbated substance-use behaviors. Key exposure pathways linking the climate crisis with substance misuse disorders or relapse include increased psychosocial stress in response to climate-driven disruptions to social, health, and economic systems, escalating rates of psychiatric morbidity or physical health complications (Vergunst et al. 2023).

In our study, the relative risk was most elevated for mood, psychoactive substance misuse, and anxiety disorders during high temperatures in the Mountains, a region characterized by traditionally lower climatological temperatures in the warm season. The increased risk of MBD-related ED visits with high temperatures is consistent with previous studies across different regions and sub-populations (Hansen et al. 2008; Peng et al. 2017; Wang et al. 2014; Yoo et al. 2021b); however, our work adds to growing evidence that populations experiencing different climatologies across geographic regions may be at greater risk for mental health effects from high temperatures (Burkart et al. 2021). Mechanistically, temperature extremes can influence mood and anxiety disorders through production and function changes in neurotransmitters like serotonin and dopamine (Ayeni et al. 2022; Karataş and Ocak 2018; Lõhmus 2018).

Interestingly, low temperatures were protective against substance misuse and mood disorders in the Mountains, associated with an increased risk for any MBD, mood, or anxiety disorders in the Piedmont region, and had no association in the Coast, suggesting the association between low temperature exposure and MBD may vary based on local geography (i.e., physiographic region) and/or underlying population. State-level results also suggest a protective effect of extremely cold temperatures (e.g., below 2.5th percentile, < 10oC), though these results could also be explained by low frequency of such extreme temperatures. The protective association between acute low temperatures and ER-MBD (RR < 1.0) that was found in the Mountains is consistent with other ER-MBD studies in Canada (Wang et al. 2014) and in China (Gao et al. 2023) that also utilized a shorter lag period. In contrast, studies that examine longer temporal periods find a larger and more significant association at more than 14 days (Yoo, Eum, Gao, et al., 2021) or 10 days (Zhang et al. 2020) with low temperature and MBD outcomes. Additional place-based investigation of mental health and extreme cold temperatures is warranted.

When looking at community-level factors like income and racial segregation (e.g., Index of Concentration at the Extremes), our study noted a stronger association at the highest temperatures (97.5th percentile) for majority-Black communities, whereas predominantly white communities had stronger associations with both cold and high temperatures. The relative risk for high temperatures was most pronounced for predominantly white communities. At the individual level, increases in temperature have shown stronger associations for mental health ED visits among white and Hispanic patients and no significant associations for Black and Asian patients (Basu et al. 2018). Our results add to this finding, showing a community-level association between high temperatures and mental health among majority white communities, which are also communities in NC associated with higher mental health burdens (Ryan et al. 2022; Sugg et al. 2022).

Elevated risk in comparatively lower-income communities (e.g., ICE: Income) were strongly associated with both high and low temperatures. The strength of this association with temperature decreased as community wealth increased. Limited research on Medicaid-paid ED visits, a potential proxy for income status, highlights a similar relationship whereby low and high temperatures lead to poorer mental health outcomes (Mullins and White 2019). In contrast, self-reported measures of mental health, as well as suicide and non-Medicaid ED visits, showed no significant moderation across income levels for high temperatures and mental health, highlighting that income may play a strong role in the mental health and temperature association for only low-income communities and individuals (Mullins and White 2019). Estimates of mental health mortality at the national level support this hypothesis with higher relative risk estimates per 1 °C for lower-income countries (Liu et al. 2021). Research in urban areas suggests heat exposure varies across and within different social geographies; inequities in excess heat exposure have consistently been identified in racially segregated and historically redlined communities (Chakraborty et al. 2023; Mitchell and Chakraborty 2018). Although a few studies have identified a null association between racialized and economic deprivation and heat stress disparities (Schinasi et al. 2022), accumulating evidence has spotlighted neighborhood poverty as a driving factor behind heat vulnerability due to diminished adaptive resources, including low tree canopy, energy poverty, and a lack of access to broadband internet that limits household access to heat warnings (Hallegatte and Rozenberg 2017; Jay et al. 2021).

High air temperatures may exacerbate pre-existing MBD conditions regardless of the potential to trigger incident mental disorders through a number of mechanistic pathways. Many medications in psychiatry increase individuals’ vulnerability to extreme temperatures by altering the body’s ability to thermoregulate (Conti et al. 2007; Martin-Latry et al. 2007). Drugs such as antipsychotics, anticholinergics, antidepressants, sedatives, and mood stabilizers commonly have side effects that impair or limit the body’s ability to sweat and/or adjust internal body temperature (Batscha 1997). These drugs are commonly prescribed to treat cases of psychosis, mood disorders, personality disorders, and anxiety disorders.

## Strengths and limitations

This study had several notable strengths. First, we evaluated the association between temperature (cold and hot) and ED visits for any MBD-related outcome, as well as specific mental disorders with a large dataset encompassing the entire state of North Carolina. This spatial extent allowed us to investigate how different geographical and climatological regions of North Carolina responded to the same percentile of temperature (2.5th and 97.5th). Second, the small-area approach that we used in creating our DLNM allowed us to scale from individual ZCTA to the pooled effect of the region (Gasparrini 2022). This methodology allows for more rural ZCTAs to be included in this study, whereas traditional DLNM approaches would have to only include more populated areas. Third, our statistical model controlled for meteorological factors to avoid potential confounding effects on the interpretation of the results. Lastly, we were able to consider multiple subgroups by examining the high and low temperature effects of sex and age groups through stratified analyses.

This study had a few limitations. First, while using a small area approach when constructing the DLNM allowed us to incorporate more ZCTAs than traditional methods would allow, sample sizes at the ZCTA level remained very small, particularly for specific sub-groups of race, limiting our ability to conduct a DLNM on these populations for individual level data. We recommend additional studies that focus on the temperature-mental health association across racial subgroups. For instance, future analysis could examine these sub-groups at a larger spatial scale (e.g., county) or higher-population locations (e.g., city) to understand the effect of extreme temperatures on this sub-population. Additionally, further work at different spatial scales can confirm our findings as our results were limited to the ZCTA scale. The mental health conditions in this study were limited to those exhibited in the emergency room, therefore, our findings are restricted to this vulnerable subpopulation for mental health, or those patients who visited the ED rather than other outpatient settings. Nonetheless, the use of emergency room visits is well-established in the literature for evaluating mental health and temperature associations (Basu et al. 2018; Niu et al. 2020; Yoo, Eum, Gao, et al., 2021) and the ED is considered a key entry point for mental health conditions in the US (Gill et al. 2017). This analysis also does not consider social factors related to mental health care (e.g., stigmatization; help seeking behaviors) nor does it consider healthcare accessibility, both of which could impact mental health-related ED visits. Lastly, the use of relative humidity is a limitation of our work and future analysis should consider metrics like dew point temperature and/or absolute humidity to evaluate how humidity moderates the temperature-mental health association.

## Conclusion

This study is among the first to comprehensively examine the relationship between temperature and MBD-related ED visits in North Carolina, USA at the community-level. Our study leveraged a daily line-level ED inpatient dataset for the entire state of North Carolina, allowing for the examination of MBD response to temperature. Exposure to high temperature was associated with an increased risk for total MBD-related ED visits at the state level and an increased risk of psychoactive substance use at the regional level. Patient age, in addition to residential, and economic segregation were identified as important drivers of exacerbating mental health events during hot or cold temperatures. Results from this study suggest regional and subpopulation differences in the temperature mental health response for those who were exposed to high and low temperatures; this knowledge can be applied across North Carolina to inform future targeted warnings and public health interventions.

## Supplementary Information

Below is the link to the electronic supplementary material. 

ESM1 (DOCX 526 KB)

ESM2 (DOCX 11.9 KB)

## Data Availability

Due to confidentiality agreements, supporting data can only be made available to bona fide researchers subject to a non-disclosure agreement. Details of the data and how to request access are available from the North Carolina Department of Health Human Services.
