# Supplementary material for: A small area analysis of acute exposure to temperatures and mental health in North Carolina
Source: Int J Biometeorol. 2025 Feb 4;69(4):805–19. doi: 10.1007/s00484-025-02858-y (PMC11947002; doi:10.1007/s00484-025-02858-y)
Supplement: Supplementary file 1 — (DOCX 526 KB) [file 484_2025_2858_MOESM1_ESM.docx]

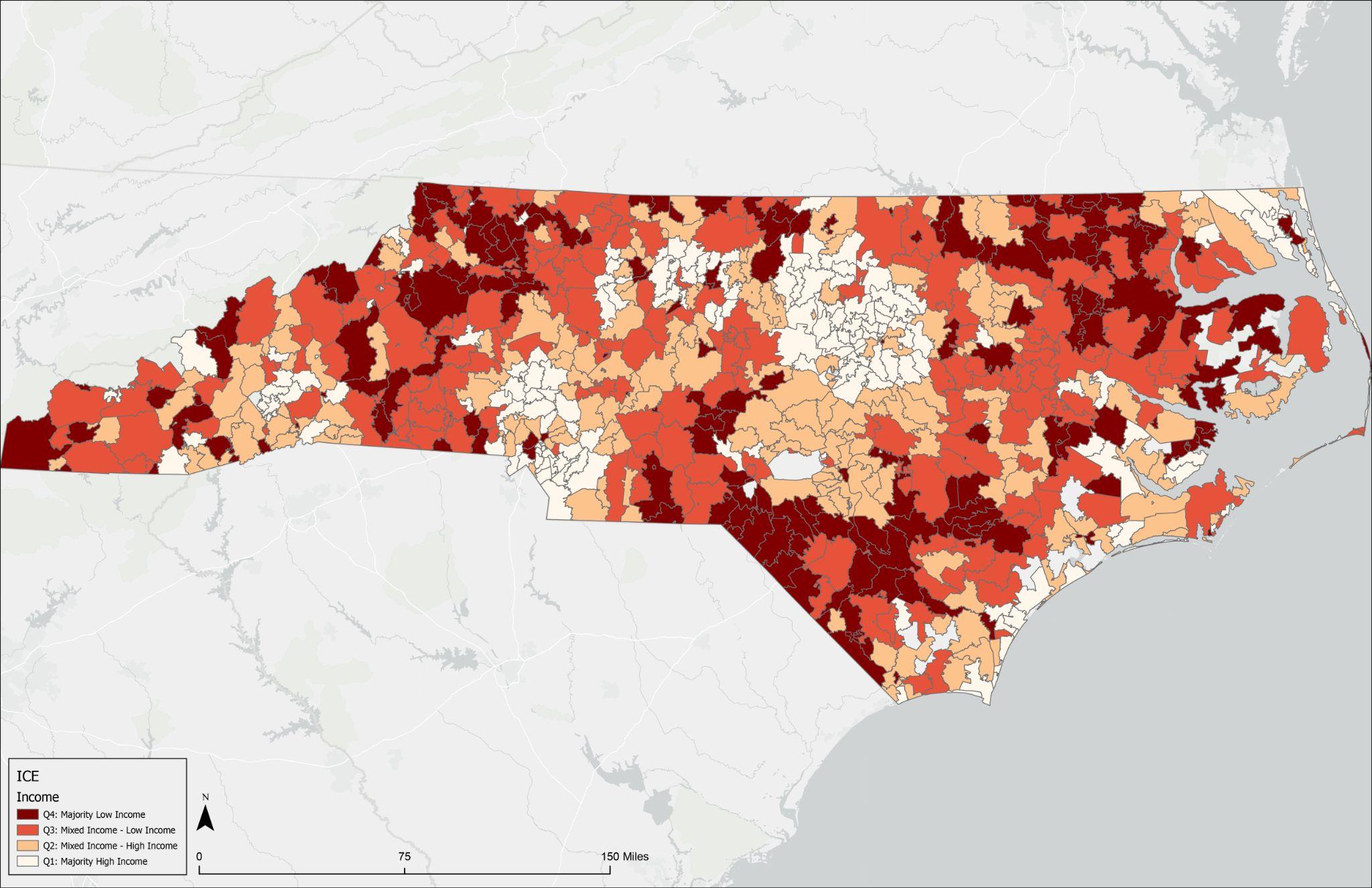

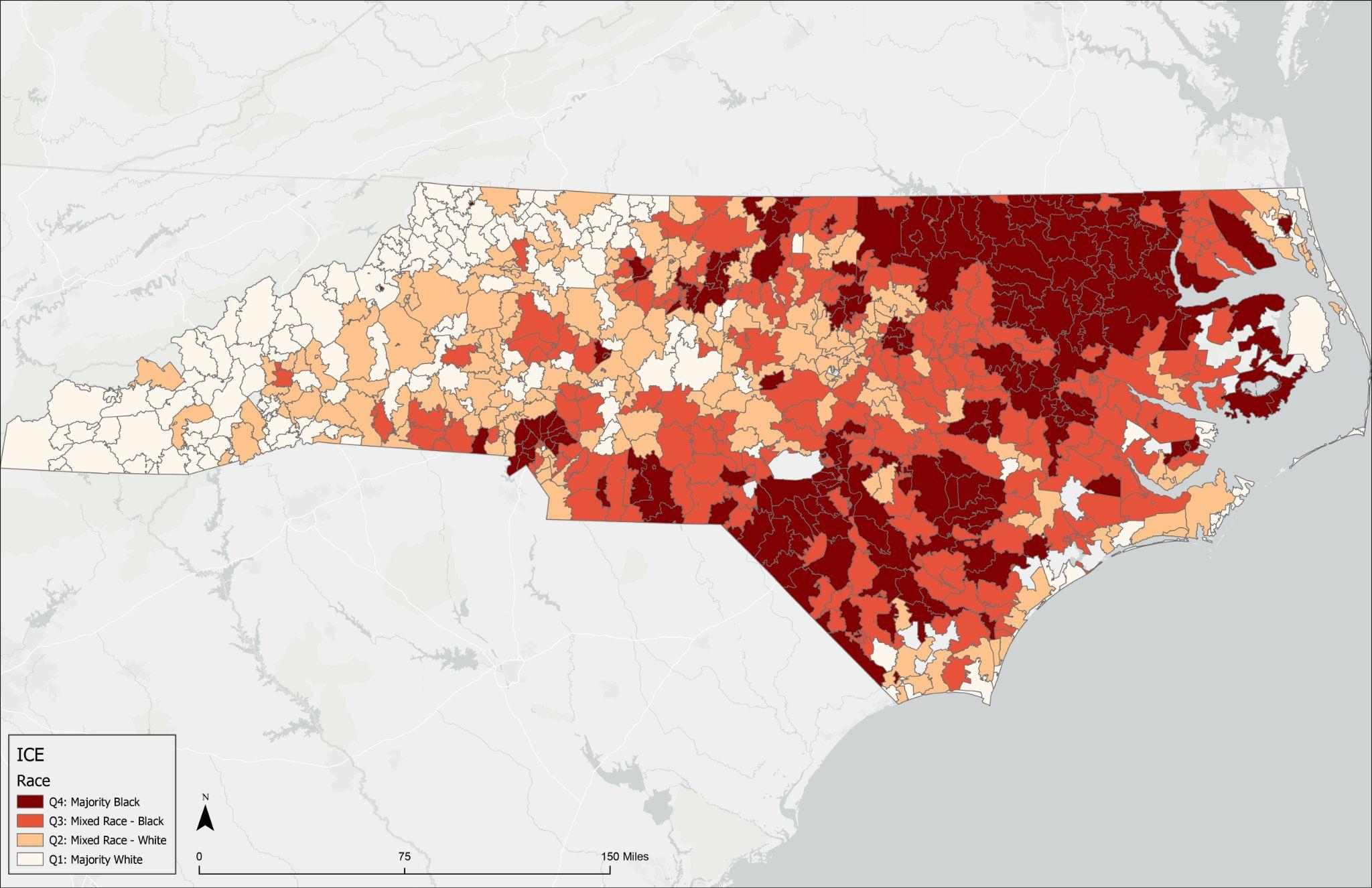


Supplemental Figure 1. Maps depicting the spatial distribution of economic privilege (ICE: Income) and racial segregation (ICE: Race) in North Carolina.
