# Supplementary material for: A small area analysis of acute exposure to temperatures and mental health in North Carolina
Source: Int J Biometeorol. 2025 Feb 4;69(4):805–19. doi: 10.1007/s00484-025-02858-y (PMC11947002; doi:10.1007/s00484-025-02858-y)
Supplement: Supplementary file 2 — (DOCX 11.9 KB) [file 484_2025_2858_MOESM2_ESM.docx]

***Supplemental tables***

Sup 1. The cumulative relative risk associated with MBD-related ER visits for total mental disorders, mental disorder subgroups, sex, and age in the Mountain, Piedmont, and Coast regions of North Carolina from 2016 to 2019 at the 2.5th and 97.5th percentile of temperature.

|  | **Mountains** | | **Piedmont** | | **Coast** | |
| --- | --- | --- | --- | --- | --- | --- |
|  | Cold | Heat | Cold | Heat | Cold | Heat |
| ***Mental disorders*** |  |  |  |  |  |  |
| Total | 0.98 (0.95-1.00) | 1.09 (1.06-1.12) | 1.02 (1.01-1.03) | 1.04 (1.03-1.05) | 1.00 (0.99-1.02) | 1.02 (1.01-1.04) |
| Substance misuse | 0.97 (0.94-0.99) | 1.13 (1.09-1.17) | 1.02 (1.01-1.04) | 1.07 (1.06-1.09) | 1.00 (0.98-1.02) | 1.04 (1.02-1.06) |
| Mood | 0.94 (0.89-0.98) | 1.09 (1.03-1.15) | 1.03 (1.01-1.05) | 1.01 (0.98-1.04) | 0.97 (0.93-1.00) | 1.03 (1.00-1.07) |
| Anxiety | 0.97 (0.93-1.02) | 1.06 (1.01-1.12) | 1.01 (0.99-1.04) | 1.02 (0.99-1.04) | 1.00 (0.96-1.03) | 1.03 (0.99-1.06) |
| ***Sex*** |  |  |  |  |  |  |
| Male | 0.98 (0.94-1.01) | 1.09 (1.04-1.13) | 1.01 (1.00-1.03) | 1.05 (1.03-1.07) | 1.02 (0.99-1.04) | 1.03 (1.01-1.06) |
| Female | 0.96 (0.93-0.99) | 1.09 (1.05-1.13) | 1.02 (1.00-1.03) | 1.03 (1.01-1.05) | 0.98 (0.96-1.00) | 1.01 (0.99-1.04) |
| ***Age group*** |  |  |  |  |  |  |
| Below 25 | 0.92 (0.86-0.98) | 0.99 (0.92-1.08) | 0.99 (0.96-1.02) | 0.97 (0.94-1.00) | 0.97 (0.93-1.01) | 0.96 (0.92-1.00) |
| 26-49 | 0.96 (0.93-1.00) | 1.12 (1.07-1.17) | 1.02 (1.00-1.03) | 1.07 (1.05-1.09) | 0.98 (0.96-1.01) | 1.03 (1.01-1.06) |
| 50-64 | 1.01 (0.96-1.06) | 1.10 (1.04-1.16) | 1.03 (1.01-1.06) | 1.07 (1.04-1.09) | 0.99 (0.96-1.02) | 1.03 (0.99-1.06) |
| Above 65 | 1.03 (0.97-1.08) | 1.04 (0.98-1.11) | 1.02 (1.00-1.05) | 0.97 (0.95-1.00) | 1.03 (1.00-1.07) | 1.02 (0.98-1.06) |
| ***ICE Race*** |  |  |  |  |  |  |
| Q1: Lowest Deprivation (Majority White) | 0.98 (0.96 - 1.01) | 1.08 (1.05 - 1.12) | 1.02 (1.01 - 1.04) | 1.04 (1.02 - 1.06) | 1.02 (1.00 - 1.05) | 1.03 (1.00 - 1.06) |
| Q2: Middle Low Deprivation | 0.99 (0.87 - 1.12) | 1.11 (0.95 - 1.31) | 1.01 (0.99 - 1.04) | 1.05 (1.02 - 1.07) | 0.99 (0.97 - 1.02) | 1.02 (1.00 - 1.05) |
| Q3: Middle High Deprivation | NA | NA | 1.02 (0.99 - 1.05) | 1.03 (1.00 - 1.07) | 1.01 (0.97 - 1.05) | 1.00 (0.96 - 1.04) |
| Q4: Highest deprivation (Majority low-income) | NA | NA | 1.06 (1.02 - 1.11) | 1.04 (1.00 - 1.09) | 0.96 (0.87 - 1.06) | 1.13 (1.02 - 1.25) |
| ***ICE Income*** |  |  |  |  |  |  |
| Q1: Lowest Deprivation (Majority high-income) | 0.98 (0.57 - 1.70) | 1.16 (0.66 - 2.05) | 0.99 (0.95 - 1.04) | 0.96 (0.91 - 1.01) | 0.97 (0.84 - 1.11) | 0.96 (0.83 - 1.10) |
| Q2: Middle Low Deprivation | 0.95 (0.86 - 1.06) | 1.06 (0.94 - 1.20) | 1.01 (0.99 - 1.04) | 1.03 (1.00 - 1.06) | 1.00 (0.95 - 1.06) | 1.02 (0.96 - 1.08) |
| Q3: Middle High Deprivation | 0.97 (0.94 - 1.01) | 1.06 (1.02 - 1.11) | 1.02 (1.01 - 1.04) | 1.05 (1.03 - 1.07) | 1.02 (1.00 - 1.05) | 1.02 (1.00 - 1.04) |
| Q4: Highest deprivation (Majority low-income) | 0.99 (0.96 - 1.03) | 1.10 (1.06 - 1.15) | 1.03 (1.01 - 1.05) | 1.05 (1.02 - 1.07) | 0.98 (0.96 - 1.01) | 1.03 (1.00 - 1.06) |
